# Prevalence of hepatitis B virus, hepatitis C virus, human immunodeficiency virus and *Treponema pallidum* infections in hospitalized patients before transfusion in Xiangya hospital Central South University, China from 2011 to 2016

**DOI:** 10.1186/s12879-018-3051-7

**Published:** 2018-04-02

**Authors:** Wei-Wei Cao, Rong-Rong Zhou, Xinghua Ou, Ling-Xi Shi, Chao-Qi Xiao, Ting-Yin Chen, Hua Tan, Xue-Gong Fan, Bi-Juan Li, Ning Li

**Affiliations:** 1 Department of Blood Transfusion of Xiangya Hospital, Central South University, 87 Xiangya Road, Changsha, 410008 Hunan Province China; 2 Department of Infectious Diseases, Xiangya Hospital, Central South University, Changsha, China; 3 Center for Disease Prevention and Control of Changsha, Changsha, China; 4 Department of Information of Xiangya Hospital, Central South University, Changsha, China; 5 Center for Disease Prevention and Control of Kaifu District, Changsha, China

**Keywords:** Transfusion-transmissible infections, Human immunodeficiency virus, Viral hepatitis, *Treponema pallidum*, Blood transfusion

## Abstract

**Background:**

Human immunodeficiency virus (HIV), hepatitis B virus (HBV), hepatitis C virus (HCV) and *Treponema pallidum* (TP) infections are considered classic transfusion-transmissible infections (TTIs). Few data are available about the prevalence of TTIs in patients before blood transfusion in China. This study aimed to investigate the seroprevalence of four TTIs among patients before blood transfusion in Xiangya Hospital Central South University, China.

**Methods:**

From 2011 to 2016, 442,121 hospitalized patients before possible blood transfusion were tested for hepatitis B surface antigen (HBsAg), anti-HCV, syphilis antibody (anti-TP) and anti-HIV.

**Results:**

Of the 442,121 patients, the overall positivity of the four TTI serum markers was 15.35%. The positive rates of HBsAg, anti-HCV, anti-HIV and anti-TP were 10.98, 1.43, 0.16 and 2.78%, respectively. TTI serum markers showed a significant difference by gender, with positive rates of 17.98% for males and 12.79% for females. The prevalence of TTI serum markers varied significantly by age. The overall co-infection rate was 0.63%, and the top three multiple infections were HBV-TP, HBV-HCV, and HCV-TP. The co-infection rates of HBV-TP and HBV-HCV showed a significant decrease from 2011 to 2016, while the rates of other co-infections remained stable.

**Conclusions:**

The prevalence of TTIs in patients before blood transfusion is much higher compared to that in blood donors in the region. The infection rates of HIV and TP increased, and the infection rate of HBsAg decreased in recent years.

**Electronic supplementary material:**

The online version of this article (10.1186/s12879-018-3051-7) contains supplementary material, which is available to authorized users.

## Background

Blood transfusion, especially component transfusion such as packed red blood cells, platelet concentrates, fresh frozen plasma, individual factor concentrates, and cryoprecipitate, has become an indispensable medical treatment for the rescue of patients. However, blood transfusion still has some limitations, and the most significant one is the potential risk of transfusion-transmitted infections (TTIs) [1, 2]. The pathogens involved in TTIs include viruses, bacteria and parasites [3]. Among them, hepatitis B virus (HBV), hepatitis C virus (HCV), human immunodeficiency virus (HIV) and *Treponema pallidum* (TP) are especially concerning because of their prolonged presence in the blood and body fluids of carriers [1–4].

With a general population infection rate of nearly 10.00% [5], the prevalence of chronic hepatitis B (CHB) is particularly serious in China. CHB and chronic HCV infection are strongly associated with liver failure, liver cirrhosis and cancer. In the early and mid-1990s in China, the anti-HCV antibody was not screened for in blood for transfusion, and this resulted in many cases of transfusion-transmitted hepatitis C [6, 7]. Acquired immune deficiency syndrome (AIDS) caused by HIV infection has become one of the world’s most serious health challenges. In 2005, 22,000 out of 75,000 newly developed HIV infections in China were acquired by transfusion of contaminated blood [8–10]. TP is an important bloodborne disease, and a resurgence of it has occurred in recent years in China [11]. Regardless of the manner of infection acquisition, timely detection and diagnosis of these TTIs is critical for the treatment of these patients and the prevention of the transmission.

The prevalence of TTIs varies greatly in different areas and populations. For instance, in western China, the prevalence rate of HIV infection in donors was 0.31% [12, 13]. While in Guangzhou and Nanjing, it was 0.02 and 0.08%, respectively [14]. Until now, most studies have focused on the positive rate of TTI serum markers among blood donors, while few studies have focused on the patients, especially these who will receive transfusion.

In the present study, we analyzed the results of tests on patients before possible blood transfusion at a comprehensive teaching hospital in the central and southern region of China and evaluated the positivity of HBsAg, anti-HCV, anti-HIV and syphilis antibodies over a 6-year period. This study provides data about the prevalence and trends of these four TTIs in a large patient population.

## Methods

The cross-sectional study was approved by “Medical Ethics Committee of the Xiangya Hospital of Centre South University” in the year 2010. Written informed consent was obtained from all the patients to allow the use of anonymized test results of their blood samples. Consent was obtained from a parent or guardian on behalf of any participants under the age of 16. Xiangya Hospital is a large-scale, comprehensive, tertiary hospital with 3500 beds and nearly 3 million outpatients and emergency patients each year. The patients at this hospital mainly come from the central and southern regions of China. From 2011 to 2016, TTI serum markers were tested in 442,121 patients who planned to receive blood transfusions, surgeries or interventional procedures at Xiangya Hospital.

### Serologic assays

Serum samples were assessed for antibodies to HIV types 1 and 2 (anti-HIV1/2), HBsAg, anti-HCV and anti-TP by chemiluminescence microparticle immunoassays (CMIA) on the ARCHITECT i2000SR (Abbott Diagnostics, Wiesbaden, Germany). All the reagents were provided by Abbott Company and were approved by the State Food and Drug Administration of China. The system provided standardized quantitative results for HBsAg, expressed in international units (IU/ml), and values exceeding 0.05 IU/ml were considered positive for HBsAg. The presence or absence of anti-HIV 1/2, anti-HCV or anti-TP in the sample was determined by comparing the chemiluminescent signal in the reaction to the cut-off signal determined from an active ARCHITECT anti-HIV1/2, anti-HCV or anti-TP calibration curve, respectively. All the results of anti-HIV1/2, anti-HCV or anti-TP are expressed as the S/CO ratio, with S/CO < 1.0 indicating a nonreactive result and S/CO ≥ 1.0 indicating a reactive result. The samples positive for anti-TP were confirmed with identical test kits. A result was considered positive only when both results were positive. Serum from patients who screened positive for anti-HIV1/2 was collected again on the second day and re-examined by CMIA and colloidal gold immunochromatographic assay (Wantai, Beijing, China). The HIV antigen was fixed on the membrane with the nitrocellulose membrane as the carrier, and the samples were migrated along the solid-phase carrier. The quality-control band of the effective test had to be developed. The positive results and the quality-control band each showed a colored stripe on the membrane antigen site. As long as one method of re-examination result was positive, the serum samples were retested to confirm the existence of HIV infection by Western blot, which was performed in the laboratory of Hunan Provincial Centers for Disease Control and Prevention (CDC) [15]. The operation methods and results of judgment strictly followed the national guideline for detection of HIV/AIDS formulated by the CDC. All reagents were of quality batches and were used within the validity period.

### Statistical analysis

Statistical analyses were conducted using SPSS 19.0 statistics software. The chi-square test was applied to assess associations between categorical variants. The chi-square test for trends was used to analyze the differences in the seroprevalence of HBsAg, anti-HCV, anti-HIV and anti-TP among patients. A *p*-value < 0.05 was considered statistically significant.

## Results

### Demographic characteristics of patients

A total of 442,121 patients were screened for serum markers of the four TTIs during the study period. Of them, 218,378 (49.39%) were male, and 223,743 (50.61%) were female. The age of the study subjects ranged from 0 to 99 years (the average age was 43.85), and patients aged from 21 to 50 years accounted for 52.91% of all the subjects. The details are shown in Table 1.

**Table 1:** Socio-demographic characteristics of screened patients from Xiangya Hospital Central South University, China, 2011–2016

Characteristic	Number of patients screened	%
Total	442,121	100.00
Gender		
Male	218,378	49.39
Female	223,743	50.61
Age		
≤ 20	53,903	12.19
21–30	71,362	16.14
31–40	75,280	17.03
41–50	87,275	19.74
51–60	70,723	16.00
61–70	55,256	12.50
≥ 71	28,322	6.40
Anti-HIV		
Positive	709	0.16
HBsAg		
Positive	48,536	10.98
Anti-HCV		
Positive	6331	1.43
Anti-TP		
Positive	12,299	2.78

### Seroprevalence of HBV, HCV, HIV and TP

The overall positivity of TTI serum markers was 15.35% (67875) and the positive rates of HBsAg, anti-HCV, anti-HIV and anti-TP were 10.98, 1.43, 0.16 and 2.78%, respectively (Table 1). Among them, 14.06% (62184) had one positive TTI serum marker, and 0.63% (2788) had two or more (Table 2).

**Table 2:** Positive rates of HBsAg, anti-HCV, anti-HIV and anti-TP among patients from Xiangya Hospital Central South University, China, 2011–2016

Year	Number of patients screened	Total	Anti-HIV	HBsAg	Anti-HCV	Anti-TP
		Number (%)	Number (%)	Number (%)	Number (%)	Number (%)
Both male and female						
2011	53,779	8911 (16.57)	67 (0.12)	6779 (12.61)	820 (1.53)	1245 (2.32)
2012	63,299	9729 (15.37)	106 (0.17)	7191 (11.36)	806 (1.27)	1626 (2.57)
2013	70,277	10,950 (15.58)	98 (0.14)	7963 (11.33)	984 (1.40)	1905 (2.71)
2014	78,709	12,177 (15.47)	123 (0.15)	8605 (10.93)	1216 (1.55)	2233 (2.84)
2015	83,888	12,263 (14.62)	144 (0.17)	8531 (10.17)	1123 (1.34)	2465 (2.94)
2016	92,169	13,845 (15.02)	171 (0.19)	9467 (10.27)	1382 (1.50)	2825 (3.06)
*P*		0.000	0.005	0.000	0.702	0.000
Male						
2011	26,697	5214 (19.53)	38 (0.14)	4182 (15.66)	438 (1.64)	556 (2.08)
2012	31,555	5745 (18.21)	77 (0.24)	4436 (14.06)	423 (1.34)	809 (2.56)
2013	34,852	6511 (18.68)	73 (0.21)	4976 (14.28)	517 (1.48)	945 (2.71)
2014	38,205	6964 (18.23)	83 (0.22)	5194 (13.60)	622 (1.63)	1065 (2.79)
2015	40,443	7003 (17.32)	99 (0.25)	5150 (12.73)	585 (1.45)	1169 (2.89)
2016	46,626	7817 (16.77)	125 (0.27)	5631 (12.08)	694 (1.49)	1367 (2.93)
*P*		0.000	0.001	0.000	0.108	0.000
Total	218,378	39,254 (17.98)	495 (0.23)	29,569 (13.54)	3279 (1.50)	5911 (2.71)
Female						
2011	27,082	3697 (13.65)	29 (0.11)	2597 (9.59)	382 (1.41)	689 (2.54)
2012	31,744	3984 (12.55)	29 (0.09)	2755 (8.68)	383 (1.21)	817 (2.57)
2013	35,425	4439 (12.53)	25 (0.09)	2987 (8.43)	467 (1.32)	960 (2.71)
2014	40,504	5213 (12.87)	40 (0.10)	3411 (8.42)	594 (1.47)	1168 (2.88)
2015	43,445	5260 (12.11)	45 (0.10)	3381 (7.78)	538 (1.24)	1296 (2.98)
2016	45,543	6028 (13.24)	46 (0.10)	3836 (8.42)	688 (1.51)	1458 (3.20)
*P*		0.112	0.805	0.000	0.279	0.000
Total	223,743	28,621 (12.79)	214 (0.10)	18,967 (8.48)	3052 (1.36)	6388 (2.86)
Age						
≤20	53,903	2112 (3.92)	45 (0.09)	1259 (2.34)	351 (0.65)	457 (0.85)
21–30	71,362	9730 (13.63)	131 (0.18)	7695 (10.78)	689 (0.97)	1215 (1.70)
31–40	75,280	13,983 (18.57)	146 (0.19)	10,697 (14.21)	1028 (1.37)	2112 (2.81)
41–50	87,275	16,911 (19.38)	166 (0.19)	12,483 (14.30)	1536 (1.76)	2726 (3.12)
51–60	70,723	12,288 (17.37)	130 (0.18)	8653 (12.24)	1253 (1.77)	2252 (3.18)
61–70	55,256	8968 (16.23)	76 (0.14)	5751 (10.41)	975 (1.76)	2166 (3.92)
≥71	28,322	3883 (13.71)	15 (0.05)	1998 (7.05)	499 (1.76)	1371 (4.84)
*P*		0.000	0.000	0.000	0.000	0.000

The positive rates of TTI serum markers were 17.98% for males and 12.79% for females (*p* < 0.001). HIV, HBV and HCV infection were more prevalent in males (*p* < 0.001). TP infection was more prevalent in females (*p* = 0.003) (Table 2).

The prevalence of TTI serum markers varied significantly by age group. The highest infection rate of HBV was found in the middle-young group (aged 31–60 years), especially in those from 41 to 50 years old (14.30%). The prevalence of HBsAg was lowest in those under the age of 20 years (2.34%). Most infections of TP were identified in aged patients. The positive rates of anti-TP significantly increased with increasing age and reached a peak (4.84%) in the age group older than 71 years. Likewise, the prevalence of HCV infection was high within those groups whose age exceeded 41 years. Overall, infection rates of HIV were relatively low, and those aged from 21 to 60 years had the highest positive rate of 0.19% (Table 2).

### Trends of Seroprevalence of HBV, HCV, HIV and TP

The positive rate of HBsAg decreased from 12.61% in 2011 to 10.27% in 2016 (*p* < 0.001), while the positive rates of anti-HIV and anti-TP increased significantly (from 0.12% in 2011 to 0.19% in 2016 (*p* = 0.005) and from 2.32% in 2011 to 3.06% in 2016 (*p* < 0.001). The positive rate of anti-HCV remained relatively steady over the 6-year study period (*p* = 0.702).

The variation trends of the positive rates of anti-TP, anti-HCV and HBsAg in male and female patients year by year were consistent. The positive rates of anti-HIV in male patients increased year by year (*p* = 0.001), while in female patients, it remained relatively steady (*p* = 0.061) (Table 2).

**Fig. 1:**
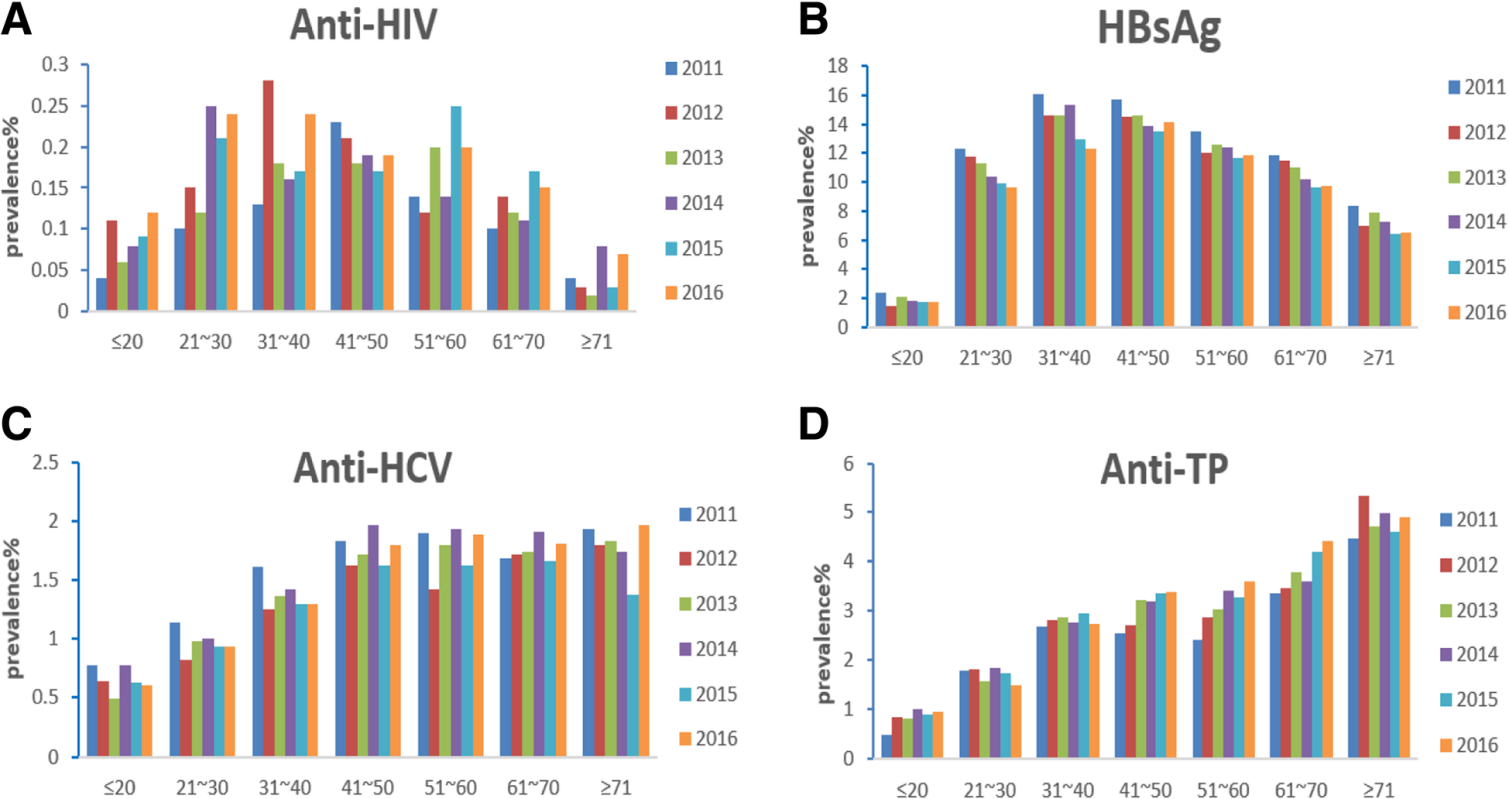
Trends of age- related positive rates of HBsAg (panel **b**), aAnti-HCV (panel **c**), Aanti-HIV (panel **a**), and Aanti-TP (panel **d**), in patients from Xiangya Hospital Central South University, China, 2011–2016

Age- and year-related distribution figures showed that the positive rates of anti-HCV and anti-TP remained steady over the 6 years studied (Fig. 1c & d). Decreased positivity of HBsAg was observed in all age groups when analyzed year by year (Fig. 1b). A significant increase in the positive rate of anti-HIV in 21–30-year-old patients was observed (*P* = 0.032) (Fig. 1a).

### Distribution of co-infections

Of all the patients screened, 2788 (0.63%) had multiple infections (Table 3). The top three multiple infections were HBV-TP, HBV-HCV, and HCV-TP, with positivity rates of 0.29, 0.18 and 0.06%, respectively. Co-infection rates of HBV-TP and HBV-HCV showed significant decreases from 0.34 to 0.23% and 0.25 to 0.14%, respectively (*p* < 0.001), while the seroprevalence of HIV-HBV showed a slight increase from 0.03 to 0.05% (*p* = 0.020). The prevalence of triple or quadruple infection was rare and remained relatively steady.

**Table 3:** Prevalence of co-infections among patients from Xiangya Hospital Central South University, China, 2011–2016

Co-infections	2011	2012	2013	2014	2015	2016	Total
	Number(%)	Number(%)	Number(%)	Number(%)	Number(%)	Number(%)	Number(%)
HBV-TP ^a^	181 (0.34)	222 (0.35)	230 (0.33)	243 (0.31)	202 (0.24)	214 (0.23)	1292 (0.29)
HBV-HCV ^a^	132 (0.25)	135 (0.21)	148 (0.21)	138 (0.18)	123 (0.15)	129 (0.14)	805 (0.18)
HCV-TP	43 (0.08)	39 (0.06)	43 (0.06)	38 (0.05)	47 (0.06)	57 (0.06)	267 (0.06)
HIV-HBV ^a^	14 (0.03)	16 (0.03)	27 (0.04)	34 (0.04)	28 (0.03)	48 (0.05)	167 (0.03)
HIV-TP	10 (0.02)	19 (0.03)	15 (0.02)	21 (0.03)	27 (0.03)	32 (0.03)	124 (0.03)
HIV-HCV	4 (0.01)	8 (0.01)	3 (0.00)	4 (0.01)	8 (0.01)	12 (0.01)	39 (0.01)
HBV-HCV-TP	14 (0.03)	5 (0.01)	16 (0.02)	6 (0.01)	5 (0.01)	5 (0.01)	51 (0.01)
HIV-HBV-TP	1 (0.00)	5 (0.01)	5 (0.01)	5 (0.01)	4 (0.01)	5 (0.01)	25 (0.01)
HIV-HBV-HCV	0	0	3 (0.00)	3 (0.00)	1 (0.00)	4 (0.00)	11 (0.00)
HIV-HCV-TP	1 (0.00)	0	2 (0.00)	1 (0.01)	1 (0.00)	2 (0.00)	7 (0.00)
HIV-HBV-HCV-TP	0	0	0	0	0	0	0
Total ^a^	400 (0.74)	449 (0.71)	492 (0.70)	493 (0.63)	446 (0.53)	508 (0.55)	2788 (0.63)

^a^: *P*_HBV-TP:_0.000(*X*^2^ = 13.709), *P*_HBV-HCV:_0.000(*X*^2^ = 21.044), *P*_HIV-HBV:_0.020(*X*^2^ = 5.426) *P*_Total_:0.000(*X*^2^ = 20.382)

## Discussion

The detection of TTI markers before blood transfusion in recipients is not only an important measure to reduce or eliminate medical disputes caused by infections through blood transfusion but also an effective way to reduce the iatrogenic infection and occupational exposure risk of doctors and nurses. Currently, available data about the prevalence of TTIs in recipients is limited, and our study supplies a relatively large amount of patient population data. Part of our data had been presented before at the 26th Annual Conference of APASL in Shanghai [16]. Of the 442,121 patients involved, 15.35% had a minimum of one positive TTI serum marker, and 0.63% had two or more. The prevalence of most TTIs in patients is higher than that in the general population and healthy donors. The positive rates of TTI serum markers in males are significantly higher than that in females.

The HBV serum epidemiology survey in China in the year 2006 indicated that the rate of HBsAg positivity was 7.18% in people aged 1–59 years [5], which showed that China has changed from a high- into an intermediate-prevalence area. In our study, the patients’ positivity of HBsAg was 10.98%, which is much higher than the 7.80% in the general population and the 0.51–1.16% in blood donors [12, 13, 17]. Seropositive rate of HBsAg was significantly higher in men than women that is consistent with previous reports. In data from a national survey in China in 2014, HBsAg positivity in people aged 1–4 years, 5–14 years and 15–29 years was 0.32, 0.94 and 4.38%, respectively. This decrease in young people was closely related to the national vaccination program implemented in 1992, which ensured that free HBV vaccination was provided for all neonates born in China [18]. In this research focused on patients, HBsAg prevalence peaked in the 41–50-year age group, and a declining trend was observed in young patients, which is in accordance with the general population. Nevertheless, CHB is still one of the most important infectious diseases in China, especially in the patient population.

Our research showed the positive rate of anti-HCV was 1.43%, which was much higher than the 0.43% reported in the general population in 2006 in China [19]. The positivity of anti-HCV in our patient group was significantly lower than 2.8%, the average level in the world, and this further supports the assertion that China is a low-epidemic area of HCV [6, 7]. The prevalence of HCV infection was high within those aged 41–70 years (1.76%). In China, blood transfusion was an important transmission route of HCV and HIV before the Blood Donation Law came into effect in 1998 [20–22]. This may partly explain the reason that HCV infection was especially high in those aged people.

China has experienced a dramatic resurgence of syphilis in recent years [11]. The total positive rate of anti-TP was 2.78%, and most infections of TP were identified in aged patients. This result was consistent with that of previous studies [23]. Approximately 18% of anti-TP positive patients aged ≥70 years had false results due to the limitations in the detection methods caused by interference by other diseases [24]. To correct this bias, some of the CMIA anti-TP-positive results were confirmed by TPPA assay in our study, but the false-positive rate of the CMIA assay was only 0.16% (Additional file 1). Therefore, the positive rate of 4.98% might be slightly higher than the real level, but the elderly infected patients, who are commonly not well educated and face the problem of economic and moral condemnation, should not be ignored [25–27]. Unlike CHB and AIDS, infection with HCV and TP is treatable. If treatment is not applied duly, some irreversible complications may lead to poor clinical outcomes. Thus, early detection and therapy in these asymptomatic patients is necessary.

The overall infection rate of HIV was 0.16% and showed an increasing trend in recent years. The most significant increases were observed in patients aged 21–30 years. In China, HIV transmission was particularly high among injection drug users (IDUs) and former plasma donors from 1985 to 2006 [9, 20, 28]. However, in recent years, sexual transmission (both heterosexual and homosexual) has become the dominant route [28]. Our study showed that the peak of HIV infections occurred in a sexually active age. In our study the infection rate of HIV in men (0.23%) was significantly higher than that in women (0.10%). Available data indicated that the HIV epidemic among men who have sex with men (MSM) has been rapidly expanding in recent years in China [29, 30]. The prevalence of TTI co-infection was 0.63% in our patient population, and its reported positivity in donors varies among areas ranged from 0.80 to 6.29% [2, 13, 31]. This is probably due to the fact that the pathogens in co-infection conditions share similar routes of transmission, including sharing needles and high-risk sexual behaviors.

The Blood Donation Law in China prohibited the use of paid blood donation and required testing for the four TTIs discussed in this study in donors and in blood products for input. As a result, volunteer donors without payment now constitute the majority of blood donors. This transformation in the composition of blood donors has been associated with a gradual decrease in the prevalence of transfusion-transmitted HCV and HIV [9, 22]. In addition, the use of clean and disposable injection equipment has helped to prevent the transmission of blood borne diseases. At present, the most concerning problem is the transmission of these diseases through sexual contact and IDUs [29].

## Conclusions

The prevalence of HBV, HCV, HIV and TP infections in patients before blood transfusion in central and south China is relatively high. The overall infection rate of HBV is high but declining in young patients, while the infection rate of HIV is relatively low but showing an upward trend. Identification of these TTI serum markers in patients before blood transfusion is strongly recommended because it could facilitate the discovery of potential infectious diseases, reduce the chance of nosocomial infection of these diseases during medical treatment and provide useful epidemiological information about TTIs in certain areas.

## Additional file

Additional file 1: Preliminary screening and validation of TP antibodies in 2016. **Table S1.** The distribution of TPPA positive specimens in different S/CO values in 2825 cases. (DOCX 30 kb)

## Abbreviations

AIDS: Acquired immune deficiency syndrome
CDC: Centers for Disease Control and Prevention
CHB: Chronic hepatitis B
CMIA: Chemiluminescence microparticle immunoassays
HBsAg: Hepatitis B surface antigen
HBV: Hepatitis B virus
HCV: Hepatitis C virus
HIV: Human immunodeficiency virus
IDU: Injection drug user
MSM: Men who have sex with men
TP: *Treponema pallidum*
TTIs: Transfusion-transmissible infections
