# Supplementary material for: Prevalence of hepatitis B virus, hepatitis C virus, human immunodeficiency virus and Treponema pallidum infections in hospitalized patients before transfusion in Xiangya hospital Central South University, China from 2011 to 2016
Source: BMC Infect Dis. 2018 Apr 2;18:145. doi: 10.1186/s12879-018-3051-7 (PMC5879580; doi:10.1186/s12879-018-3051-7)
Supplement: Supplementary file 1 — Preliminary screening and validation of TP antibodies in 2016. Table S1. The distribution of TPPA positive specimens in different S/CO values in 2825 cases. (DOCX 30 kb) [file 12879_2018_3051_MOESM1_ESM.docx]

**Preliminary screening and validation of TP antibodies in 2016**

**Method**: Totally 92169 cases from pre-transfusion patients were tested by CMIA on the ARCHITECT i2000SR (ARCHITECT Syphilis TP Abbott Laboratories, Wiesbaden, Germany) in the year 2016**.** The screening positive samples of anti-TP were confirmed by Treponema pallidum particle agglutination test (TPPA). Serodia TPPA (Fujirebio, Tokyo,Japan) which are agglutination-based assays. For the Serodia TPPA the agglutination pattern was inspected and the results expressed as titers.

**Result:** Totally 2825 serum samples were positive of anti-TP by CMIA, and screening positive rate was 3.07%. Among them, 2680 positive serum samples were confirmed by TPPA assay and confirm positive rate was 2.91%. Compared with the TPPA assay, the false positive rate of the CMIA was 0.16%, and the positive predictive value was 94.87%. In the samples with a *S/CO* value between 1 and 2, the confirmed TP positivity was 72.8%, while in the sample with a *S/CO* value of more than 5, the confirmed positivity of TP was 100% tested with TPPA.

**Table 1 The distribution of TPPA positive specimens in different S/CO values in 2825 cases**

| **S/CO** | **Total (n)** | **Positive (n)** | **Negative (n)** | **Positivity (%)** | **X^2^** | **P** |
| --- | --- | --- | --- | --- | --- | --- |
| **1～2**  **2～3**  **3～4**  **4～5**  **5～8**  **＞8** | 404  157  129  119  348  1668 | 294  138  119  113  348  1668 | 110  19  10  6  0  0 | 72.8  87.90  92.24  94.96  100  100 | 14.61  1.47  0.75  14.02  --- | 0.000^*^  0.231  0.392  0.000^*^  --- |
| **Total** | 2825 | 2680 | 145 | 94.87 | 138.26 | 0.000^*^ |
